# Prevention of altitude illness in adults: preparing people for travel to high altitude

**DOI:** 10.1136/bmj-2026-100532

**Published:** 2026-09-02

**Authors:** Sudeep Adhikari, Rudy Zimmer, Hermann Brugger, Nischal Bajracharya, Buddha Basnyat

**Affiliations:** 1 Oxford University Clinical Research Unit—Nepal, Lalitpur, Nepal; 2 Richmond Square Medical Centre, Calgary, Canada; 3 Institute of Mountain Emergency Medicine, Eurac Research, Bolzano, Italy; 4 Patient author, Kathmandu, Nepal

**What you need to know**

- Support individuals to pursue safe and responsible high altitude travel by offering and signposting to sources of accurate and practical advice on prevention and recognition of altitude illness.
- Understand that tools exist to stratify altitude illness risk, and that a high level of baseline physical fitness and younger age are not protective against altitude illness.
- Risk can be managed by modification of travel plans and, in some cases, pharmacological prevention.

Altitude illness is a collective term for syndromes that can occur in unacclimatised people who travel to destinations higher than 2500 metres above sea level.^1,2,3^

Altitude illness is potentially life threatening; however, it is preventable and manageable with adequate pre-travel planning and preparation. Here, we offer guidance on how to advise patients regarding travel to altitudes higher than 2500 m above sea level, and when to consider directing patients to a travel medicine expert with altitude illness experience. The recommendations are based on 2024 Wilderness Medical Society (WMS) guidelines,^1^ the 2024 update of the 2012 Union Internationale des Associations d’Alpinisme (UIAA) Medical Commission guidelines,^2^and the authors’ clinical experience.

## What is altitude illness and who might be affected?

Inform anyone requesting advice regarding travel to high altitudes (box 1) about the signs and symptoms of altitude illness and how to recognise them.

Box 1 **Frequently visited high altitude destinations^4^ ^26^**

- High altitude (2500-3500 m): Ski resorts in the Colorado Rockies, US and European Alps; high altitude cities with international airports such as Bogota, Quito, Cusco, La Paz (Andes mountains), and Lhasa (Tibetan plateau).
- Very high altitude (3500-5500 m): Trekking to Everest base camp or completing the Annapurna circuit over Thorong La in Nepal, or travel across the Altiplano plateau in Andes, South America.
- Extremely high altitude (>5500 m): Climbing Mount Kilimanjaro in Tanzania (the highest peak on the African continent at 5900 m) or Mount Aconcagua in Argentina (the highest peak in the Americas at 6961 m).

People with a history of altitude illness, or who ascend rapidly (more than 500 m per day, box 2) to high altitude environments are at the greatest risk of developing altitude illness.^1^ ^3^This could include recreational trekkers, professional mountaineers, support teams (eg, porters carrying luggage), and those ascending for work, for example miners and military personnel.^4^ ^5^ ^6^

Box 2 **Definitions of commonly used mountain medicine terms^1^ ^2^**

- Sleeping altitude: The altitude above sea level at which an individual sleeps or plans to sleep overnight. (WMS and UIAA guidelines consider sleeping altitude more relevant in determining risk of altitude illness than the maximum altitude reached during the daytime).
- Sleeping altitude gain: The increase in sleeping altitude between two consecutive nights, expressed in metres. For example, if a traveller spends one night at an altitude of 3200 m and ascends over the day to an altitude of 4000 m to spend the subsequent night, then the sleeping altitude gain for that day is 800 m.
- Rate of ascent: The sleeping altitude gains in metres between two consecutive nights during the ascent, when travelling above 3000 m above sea level. The suggested safe ascent rate in WMS and UIAA guidelines is 300-500 m per day.
- Rapid ascent: A rate of ascent that is more than 500 m sleeping altitude gain between two consecutive nights of high altitude travel. This increases the risk of altitude illness. For example, if a traveller spends the night at an altitude of 3200 m and the subsequent night is spent at an altitude lower than 3700 metres above sea level, this would be a safe rate. Spending the subsequent night at an elevation above 3700 m would increase the risk of altitude illness.
- Acclimatisation: The physiological adaptation to the lower atmospheric air pressure (and reduced availability of oxygen) at higher altitudes. One way of achieving acclimatisation at high altitude is sleeping one or more extra days at the same elevation above sea level, after every 1000 m gain of the sleeping altitude. Note: trekking operators will often build an extra day into the itinerary for every 2-3 days of gain to allow for acclimatisation, assuming a rate of ascent of 300-500 m per day.
- Hypobaric hypoxia: Hypoxia (lower tissue oxygen supply) that occurs due to lower partial pressures of oxygen in the alveoli, resulting from decreased barometric pressures at high altitudes. The concentration of oxygen (about 21%) in air remains the same at sea level and high altitude, but the availability of oxygen for breathing drops at higher altitude owing to a drop in its partial pressure (as per Dalton’s Law).

The spectrum of altitude illness ranges from symptoms of acute mountain sickness (AMS) to life threatening high altitude cerebral oedema (HACE) and high altitude pulmonary oedema (HAPE) (table 1).^1^ ^3^ ^7^ ^8^ Peripheral oedema can occur at high altitudes, but is not specific to altitude illness.^9^AMS, HACE, and HAPE are all associated with hypobaric hypoxia (box 2).^10^ ^11^AMS and HACE involve cerebral pathophysiology resulting from increased cerebral blood volume and intracellular oedema in response to the hypoxia,^10^ and HAPE is a form of non-cardiogenic pulmonary oedema due to exaggerated hypoxic pulmonary vasoconstriction and elevated pulmonary artery pressure.^11^ AMS may occur in up to 25% of people with rapid ascent above 2500 m.^4^ HACE may occur in up to 1% of travellers at 4000-5000 m, and HAPE in up to 6% at 4500 m.^12^

**Table 1:** Clinical features and prognoses of altitude illnesses

Acute mountain sickness (AMS)	High altitude cerebral oedema (HACE)	High altitude pulmonary oedema (HAPE)
Headache + any of the following:^1^ • Nausea/vomiting • Fatigue • Lassitude (lack of energy) • Dizziness Some travellers may develop headaches alone, which is classified as “high altitude headache” by some experts.^7^ ^19^ Prognosis: excellent with cessation of ascent, however HACE may develop if ascent continues^4^	Deterioration of severe AMS symptoms: • Ataxia (unable to do the tandem gait—heel to toe—walk)^1^ ^2^ • Severe lassitude^1^ • Altered mental status^1^ • Slurred speech^1^ Features are similar to alcohol intoxication^4^ Progresses rapidly: coma and death can develop within 12-24 hours if not promptly treated and descent initiated^4^	• Dyspnoea (shortness of breath) on mild exertion—out of proportion with previous days’ experiences at high altitude or with experiences of fellow travellers at the same elevation^1^ • Clear drop in exercise capacity from normal baseline^1^ • Non-productive cough, fatigue, weakness, gurgling sensation in the chest^1^ Progresses to dyspnoea at rest and a productive cough with pink frothy sputum^1^ Mortality: up to 50% if left untreated.^12^Aided descent is required immediately^1^ ^2^

When travelling to altitudes >2500 m, if symptoms suggestive of altitude illness (table 1) develop, it should be considered altitude illness until proved otherwise and we advise patients to cease further ascent for mild or moderate AMS symptoms or to descend for severe AMS symptoms, HACE, or HAPE and seek medical attention.

### Symptoms on arrival at high altitude destinations

Improved access and transportation have enabled travel to high altitude environments without acclimatisation. Symptoms of altitude illness typically appear during or after the first night at such locations, and not immediately on arrival; however, experiencing mild lightheadedness or tachypnoea (increased respiratory rate) on arrival are normal responses to initial exposure to hypobaric hypoxia.

## How are people assessed before high altitude travel?

Ideally, individuals considering travel to high altitude regions would seek advice prior to booking the trip. To stratify health risk, including for altitude illness, clinicians may consider assessment of general fitness, capacity to undertake increased physical exertion, and capacity to tolerate hypoxia. It is important that clinicians advise their patients that physical fitness is not protective against altitude illness. Intense physical exertion (increased oxygen demand over baseline needs) increases risk of altitude illness, irrespective of physical fitness, and extra acclimatisation days help to decrease the exertion during the trip.

### Initial assessment

WMS guidelines^1^ and the UIAA Medical Commission^2^ recommend asking the following questions:

- Does the person have comorbidities and are these medical conditions currently stable?
- Do they have a history of known or suspected altitude illness?
- Discuss travel plans, including: destinations, maximum sleeping altitudes, possibilities for descent by at least 500 m per day, medical evacuation plans, modes of travel, and planned activities. If available, ask for trekking itineraries, which can help assess altitude illness risk.
- Discuss expected exertion during the trip.
- Are extra acclimatisation days (box 2) built into the itinerary? What local medical facilities will be available during travel, including medical personnel with the travel team, if any?
- Has adequate travel medical insurance been purchased, including insurance that covers in-country treatment, as well as medical evacuation from higher altitude remote locations to lower altitude urban locations with adequate medical facilities?

### Stratifying risk of altitude illness

Validated tools or resources such as given in 2024 WMS guidelines can be used to stratify risk of altitude illness.^1^ ^2^ ^4^Consider the factors summarised below and in table 2 when stratifying risk of altitude illness:

**Table 2:** Summary of the risk stratification scheme, as recommended by the WMS 2024 guidelines^1^

Risk of developing altitude illness	Risk criteria
**Low risk**	All of the following: • History of no or mild AMS (in those with prior travel to high altitude) • Will sleep at altitude <2800 m on day 1 of the high altitude travel • Will not ascend more than 500 m per day above 3000 m • Will have one extra day for acclimatisation after every 1000 m of ascent
**Moderate risk**	Any of the following, without any high risk features: • History of moderate or severe AMS • Will sleep at altitude 2800-3500 m on day 1 • Will ascend more than 500 m per day above 3000 m, but has extra days for acclimatisation after every 1000 m of ascent
**High risk**	Any of the following: • History of HACE/HAPE • Will sleep at altitude >3500 m on day 1 • Will ascend more than 500 m per day above 3000 m, without keeping any extra day for acclimatisation after every 1000 m of ascent

AMS=acute mountain sickness; HAC=high altitude cerebral oedema; HAPE=high altitude pulmonary oedema

#### Rate of ascent

This is the most important risk factor for developing altitude illness. The patient’s travel itinerary should provide the information required to inform this assessment. Consider the mode of transportation and time to be spent at high altitude, including the day-to-day elevation profile. The risk for developing altitude illness increases if:

- Sleeping altitude is planned at >2800 m on the first day (eg, if flying into a high altitude city)
- The person undertakes rapid ascent of more than 500 m per day of sleeping altitude gain
- There are no extra days planned for acclimatisation for every 1000 m of sleeping altitude gain.

When there is uncertainty, consider advising the patient to consult a travel medicine expert with experience in altitude illness.

#### Previous high altitude experience

Ask about previous travel to high altitudes, and specifically any history of altitude illness and its management. A history of moderate or severe AMS, HACE, or HAPE implies the same moderate or high risk of developing altitude illness in a future high altitude trip.

If signs and symptoms were severe despite appropriate pharmaceutical prevention or treatment for HACE or HAPE, advise the individual not to return to high altitudes.^2^

For individuals who have not travelled to high altitude in the past, we advise clinicians to assume that some degree of AMS, HACE, and/or HAPE may occur at the destination, depending on the characteristics identified in table 2, where the WMS risk stratification scheme is summarised.

If individuals are identified as being at moderate or high risk, offer advice (eg, adjust travel plans to reduce risk, educate on signs and symptoms of altitude illness) and consider preventive measures, including medication. Advise patients to see a travel medicine expert with high altitude experience if there will be no medical personnel on the travel team.

### What about pre-existing health conditions?

While cardiopulmonary and other comorbidities are not formally recognised risk factors for altitude illness, we suggest assessing whether comorbidities might cause complications unrelated to altitude illness.

Recommendations for pre-travel management of people with comorbidities can be found in the 2023 UIAA guidelines for travellers with diabetes,^13^ the 2018 European joint guidelines on travellers with cardiovascular conditions,^14^and the *CDC Yellow Book: Health Information for International Travel*.^4^

These include: encouraging patients to carry their regular medications (including insulin with adequate insulation) with spare supplies; carry necessary rescue medicines for spontaneous exacerbations of existing conditions; monitor chronic disease status (blood glucose, blood pressure, etc, as required) at the usual frequency; and take extra care with general preventive measures against foodborne/waterborne/respiratory pathogens if patients are immunocompromised.^15^ Patients with pre-existing medical conditions should consider consulting their specialist for advice. Those with reduced exercise capacity might wish to discuss their fitness for high altitude travel with a travel medicine expert who has altitude illness experience. Advise patients with following severe medical conditions not to travel to high altitude (box 3) because hypobaric hypoxia can lead to life threatening complications including death in these patients.^4^ ^16^

Box 3 **Contraindications to high altitude travel^6,15^**

- Advanced restrictive or obstructive lung diseases
- Decompensated heart failure
- Myocardial infarction or stroke within past six months
- Unstable angina
- Poorly controlled seizure disorder
- Pulmonary hypertension
- Sickle cell disease
- High risk pregnancy
- Untreated, high risk cerebrovascular abnormality (aneurysm or AV malformation)

## When is pharmaceutical prevention considered?

Medication may be considered to reduce the likelihood of developing altitude illness. WMS and UIAA guidelines recommend acetazolamide or dexamethasone for prevention of AMS/HACE in people with moderate or high risk for AMS/HACE. 1 2 4 17WMS and CDC guidelines recommend nifedipine, phosphodiesterase-5 (PDE-5) inhibitors, or dexamethasone for prevention of HAPE only in people with a history of HAPE.^1^ ^4^

In most European countries, including the UK, acetazolamide, dexamethasone and nifedipine are not licensed for the prevention of altitude illness. However, WMS guidelines support their prescription for off-label use, as indicated below.^1^In the UK, medicines prescribed in anticipation of altitude illness during travel would not normally be provided on an NHS prescription. GPs who are competent to assess the traveller and prescribe these medicines may issue a private prescription; where the clinician does not feel competent to do so, advice from or referral to a clinician with relevant expertise should be considered.

### Prevention of high altitude headaches

A 2010 randomised controlled trial in Nepal of 265 healthy trekkers found that incidence of high altitude headache was lower with ibuprofen 600 mg three times daily (27.5%) or acetazolamide 85 mg three times daily (27.1%) compared with placebo (45.3%),^18^and WMS guidelines suggest that oral ibuprofen 600 mg three times daily may be considered for the prevention of high altitude headaches.^1^ ^19^

### Prevention of AMS and HACE for people with moderate or high risk for AMS/HACE 1 2 4 17

Acetazolamide is recommended as the first line medication for this indication across both WMS and UIAA guidelines. A 2017 Cochrane review, Interventions for preventing high altitude illness, found moderate quality evidence to suggest acetazolamide reduces the risk of AMS (risk ratio (RR) 0.47, 95% confidence interval (CI) 0.39 to 0.56), and of HACE (RR 0.32, 95% CI 0.01 to 7.48).^17^ WMS and UIAA guidelines recommend starting this the night before ascending to a sleeping altitude above 2800 m.^1^ ^2^ After reaching the highest elevation on the itinerary, people who immediately descend can stop taking the drug after starting the descent. If the plan is to remain at the high altitude for several days, advise the patient to continue the prophylaxis for 2-4 days after reaching and remaining at that maximum altitude, or until starting descent, whichever is earlier:

- For moderate risk ascent: 125 mg dose orally every 12 hours.^1^
- For high risk ascent: consider doubling the dose (to 250 mg every 12 hours).^1^

If acetazolamide is contraindicated (eg, the patient has a history of anaphylaxis to sulphonamides or a history of Stevens-Johnson syndrome), WMS guidelines recommend considering oral dexamethasone:

- 2 mg dose orally every six hours, or
- 4 mg dose orally every 12 hours.

Tapering is not required unless used for more than seven days.

However, the 2017 Cochrane review did not show benefit from using dexamethasone (RR 0.60, 95% CI 0.36 to 1.00, with low quality of evidence), noting that included studies did not report on HACE.^17^

### Pharmaceutical prevention of HAPE in people with a history of HAPE^1^ ^4^

For HAPE prophylaxis, WMS guidelines recommend that people start medication 24 hours prior to ascent, and continue medication until initiating descent. For those who plan to stay at high altitude, advise them to continue the medication for 4-7 days after reaching the maximum elevation or until descent is initiated, whichever is earlier.

Nifedipine extended release should be considered first line according to the WMS guidelines^1^:

- 30 mg dose orally every 12 hours.^20^

This is supported by a 1991 randomised placebo controlled trial of 21 mountaineers with a history of HAPE where it was found that one of the 10 subjects who received nifedipine, and seven of the 11 subjects who received placebo, developed HAPE during a new hike to 4559 m.^21^

Consider a phosphodiesterase-5 (PDE-5) inhibitor as a second choice, such as^2^:

- Tadalafil: 10 mg dose orally every 12 hours, or
- Sildenafil: 50 mg dose orally every 8 hours.

As a third choice, consider dexamethasone as follows^2^:

- 8 mg dose orally every 12 hours.

## What further advice can be offered to patients?

Advise individuals to be aware of, and be able to identify, the symptoms of AMS, HACE, and/or HAPE (ttable able 1) in themselves and fellow travellers. Encourage them to do their own reading prior to travel. A 2022 Delphi study identified 28 essential learning objectives for travellers to high altitude.^22^ We recommend resources such as Travel Health Pro (https://travelhealthpro.org.uk/diseases/altitude-illness), which is produced by the National Travel Health Network and Centre (NaTHNaC). We advise against using pulse oximeters for trying to diagnose or confirm altitude illness^23^ as there is no evidence that oxygen saturation readings correlate with the presence of altitude illness. Some individuals may have lower readings without signs and symptoms, while others may have higher readings with severe signs and symptoms. Confusion over oximeter readings may lead to unnecessary delays in descent and self-treatment.

Treatment of acute altitude illness, including self-treatment, is beyond the scope of this paper.^24^ The authors recommend that clinicians advise travellers to stop ascent for mild and moderate AMS symptoms and immediately to descend for severe AMS, HACE, or HAPE symptoms, and to use supplemental oxygen if immediate descent is not possible.^1^ ^2^ Clinicians may consider prescribing acetazolamide (250 mg every 12 hours for AMS) and dexamethasone (4 mg every six hours for AMS and 8 mg once then 4 mg every six hours for HACE until symptoms subside) for travellers with moderate and high risk of altitude illness, for emergency use at high altitudes, in line with CDC guidance.^4^

### General advice on preventing altitude illness

Explain that being physically fit or being younger in age are not protective against altitude illness. In our experience, younger, fitter adult travellers may put themselves at greater risk compared with older less fit travellers because of the greater potential for rapid ascent rates with few extra acclimatisation days.^1^ ^2^ ^4^

Advise patients to:

- Follow a low risk gradual ascent profile as given in table 2 1 2 4
- Sleep at altitude <2800 m on day 1 of the high altitude travel
- Do not ascend more than 500 m per day above 3000 m
- Have one extra one day for acclimatisation after every 1000 metres of ascent
- Remain well hydrated for the duration of travel, as dehydration can mimic altitude illness
- Avoid drinking alcohol for the duration of high altitude travel^2^
- Consider pre-acclimatisation before travel. Repeated exposures to normobaric hypoxia at low altitudes (using hypoxic tents) in the weeks or months before high altitude travel is gaining popularity in mountain climbing. A 2025 survey of 385 mountaineers who had travelled to mountains >6000 m in altitude in the preceding 20 years found that 24.6% had used a hypoxic training system.^25^ Evidence to support pre-acclimatisation is lacking, however, and for many travellers, it may not be practical.

### What management might be needed on returning to low altitudes?

As symptoms typically subside once the individual has descended to lower altitudes, further investigation is not usually needed.^1^ ^5^ If symptoms have not subsided on return to low altitude, consider alternative causes (box 4) and refer as appropriate.^1^ ^4^ ^5^On returning home, GPs should consider documenting medical events that occurred better to inform future high altitude travel plans. We also suggest reassessing comorbidities and reviewing any complications that occurred.

Box 4 **Differentials to consider if symptoms persist on return to low altitudes**

- **AMS**: alcohol hangover, carbon monoxide poisoning, dehydration, exhaustion, hyponatraemia, migraine
- **HACE**: intracranial lesions, seizure disorder, hyponatraemia, drug intoxication, hypoglycaemia, hypothermia, stroke
- **HAPE**: anxiety attack, bronchospasm, myocardial infarction, heart failure, pneumonia, pulmonary embolism, pneumothorax, mucus plugging

**Table:** Example Kilimanjaro trek itinerary

Day	Travel	Sleeping altitude gain	Risk stratification
1	Trek from 2390 m to sleeping altitude of 2785 m	395 m	High risk for developing altitude illness: Daily gain in sleeping altitude on day 2 and day 6 is more than 500 m with no extra days for acclimatisation after ~1000 m of overall sleeping altitude gain
2	Trek to sleeping altitude of 3505 m	720 m	
3	Trek to sleeping altitude of 3895 m	390 m	
4	Trek to sleeping altitude of 3985 m	90 m	
5	Trek to sleeping altitude of 4035 m	50 m	
6	Trek to sleeping altitude of 4660 metres	625 m	
7	Summit attempt: Hike to 5895 m, then descend to sleeping altitude of 3105 m	Summit attempt, gain of 1235 m followed by a delayed descent (2790 m) after several hours above sleeping altitude	High risk of developing altitude illness during summit attempt with gain over 1000 m with significant exertion, and delayed descent
8	Descend to 1630 m		No further risk

**Patient perspective**

We took the eight day Lemosho Route trek to the summit of Mount Kilimanjaro (5895 m), which is one of the highest non-technical climbs in the world.

On the seventh day, we rose from Barafu Camp (4660 m) at nearly midnight. We had slept only three short hours, but I felt surprisingly well. Awake, steady, ready for the summit.

I’ve always been a conversational walker—who talks and strides in sync, sharing thoughts with every footfall. And I walk fast.

It was just my wife, me, two guides, and a porter. When we began the final ascent to the summit, the guides kept reminding us to walk slowly.

Hours passed and as we neared 5000 m, I had a severe headache, then fatigue came in waves. I began to pause—first every few minutes, then every few steps. Soon, I had to pause to breathe with every single step.

I thought it was the lack of sleep. But my wife, who knew my pace and energy, saw something else entirely. “This is not exhaustion,” she said firmly. “This is altitude sickness. We need to turn back.” I agreed.

To my surprise, within just half an hour of descending, I began to feel better and lighter. The headache was gone. My breathing was much easier and my strength felt restored. I was myself again.

I was aware of altitude sickness, but I had thought that a physically fit person like me would not develop it. Had I consulted my GP or travel medicine doctor before the trek, they would have advised me preventive measures and I could have completed the summit.

**Education into practice**

- How would you assess the risk of altitude illness in someone planning to travel to high altitude?
- What altitude illness symptoms do you advise patients to be able to recognise?

**How patients were involved in the creation of this article**

SA invited patient author NB to contribute his experience of high altitude illness (patient perspective). NB also reviewed the whole draft and approved the final manuscript before submission.

Based on external patient reviewers’ comments, amendments were made in prevention and treatment sections to improve clarity of the recommendations.

**How this article was created**

PubMed was searched using the MeSH terms: “(diagnosis, treatment, prevention, guideline) and (altitude illness).” The Cochrane Database was searched for related systematic reviews. Relevant guidelines, reports, and educational material from the Wilderness Medical Society, Union Internationale des Associations d’Alpinisme Medical Commission, Centers for Disease Control and Prevention, and relevant reviews on high altitude illness were examined.

## Notes

Contributorship and the guarantor: SA and BB conceived the article and are guarantors. SA, RZ, HB, and BB conducted the literature review. SA wrote the first draft. RZ, HB, and BB revised the draft and helped with tables and boxes. BB was the contact person for patient involvement. NB was the patient co-author and contributed by sharing his personal story. All authors reviewed and approved the final manuscript.

Provenance and peer review: not commissioned; externally peer reviewed.
